# Relationship between pharyngeal or esophageal reconstruction and esophageal pressure after swallowing

**DOI:** 10.1002/cnr2.1619

**Published:** 2022-04-05

**Authors:** Hiroki Umezawa, Mariko Umezawa, Yu Hokazono, Takeshi Matsutani, Rei Ogawa

**Affiliations:** ^1^ Department of Plastic, Reconstructive and Aesthetic Surgery Nippon Medical School Tokyo Japan; ^2^ Department of Gastroenterology Nippon Medical School Tokyo Japan; ^3^ Department of Surgery Nippon Medical School Tokyo Japan

**Keywords:** esophageal high‐resolution manometry, esophageal reconstruction, food‐transport dynamics, head‐and‐neck reconstruction, pharyngeal reconstruction

## Abstract

**Background:**

High‐resolution manometry, which measures esophageal luminal pressure changes after swallowing, could shed more light on food‐transport dynamics after pharyngeal/esophageal reconstruction. This prospective cohort study assessed the influence of two head‐and‐neck and esophageal tumor‐resection and reconstruction approaches on esophageal pressure.

**Methods:**

The cohort consisted of 17 patients who underwent esophageal/pharyngeal resection/reconstruction for cancer and then participated in postoperative high‐resolution manometry. Five healthy controls also underwent manometry for comparison.

**Results:**

Partial pharyngectomy with patch grafts associated with smooth and continuous esophageal/pharyngeal movement. By contrast, surgery that removed the thoracic esophagus led to complete loss of peristalsis and poor food transport.

**Conclusions:**

High‐resolution manometry effectively characterized the changes in food‐transport dynamics caused by pharyngeal/esophageal resection/reconstruction. These findings suggest that continuous and smooth movement of the pharynx and esophagus is important for swallowing and high resolution manometry could be useful in patients after pharyngeal/esophageal resection/reconstruction.

## 1 INTRODUCTION

Although the complication rate and prognosis of head and neck or esophageal (H&N‐E) cancer have improved due to technological advances in resection and reconstruction,^1^, ^2^, ^3^, ^4^ H&N‐E cancer surgery frequently results in postoperative swallowing dysfunction (60% of cases at two postoperative years).^5^ This is particularly true for patients who require reconstructive surgery after the resection.

While X‐ray videofluoroscopy^6^ is widely employed for examining swallowing function after H&N‐E reconstruction, this method does not assess the dynamic changes in esophageal luminal pressure that occur during swallowing. Its ability to identify the effects of surgery on food‐transport dynamics is thus limited. By contrast, this information can be captured by high resolution manometry (HRM) using a multi‐sensor catheter.

This prospective cohort study aimed to determine whether HRM can accurately measure the effects of various resection/reconstruction surgical approaches on pharyngeal/esophageal food‐transport dynamics.

## 2 MATERIALS AND METHODS

### 2.1 Ethics statement

This study adhered to the principles of the Declaration of Helsinki and was approved by the Institutional Review Board of Nippon Medical School Hospital (Approval No. 26‐06‐380). All patients and control subjects consented verbally and in writing to undergo HRM.

### 2.2 Patient inclusion and exclusion

The cohort consisted of all patients who (a) underwent larynx‐preserving pharyngectomy/esophagectomy for H&N‐E cancer followed by pharyngeal/esophageal reconstruction at Nippon Medical School Hospital (Tokyo, Japan) between April 2012 and November 2018 and (b) agreed to undergo HRM during a hospital visit after surgery. Patients were excluded if they were uncooperative or completely unable to take food or liquid orally. Five healthy subjects were also enrolled in the study as controls.

### 2.3 Surgical procedures

Two larynx‐preserving surgery and reconstructive approaches were employed with the eligible patients, as follows. (a) Larynx‐preserving esophageal resection and microsurgical reconstruction (designated LPE): the larynx was preserved and the thoracic and cervical esophagus were resected. The esophagus was reconstructed by gastric‐pull‐up and free‐jejunal transfer or by supercharged‐pedicled intestine transfer. (b) Partial pharyngectomy followed by reconstruction with a free‐tissue patch graft including the jejunum or forearm (designated partial pharyngectomy + patch): the tumor was resected with a sufficient margin but most of the larynx and hypopharynx could be preserved.

### 2.4 Esophageal manometry

**FIGURE 1:**
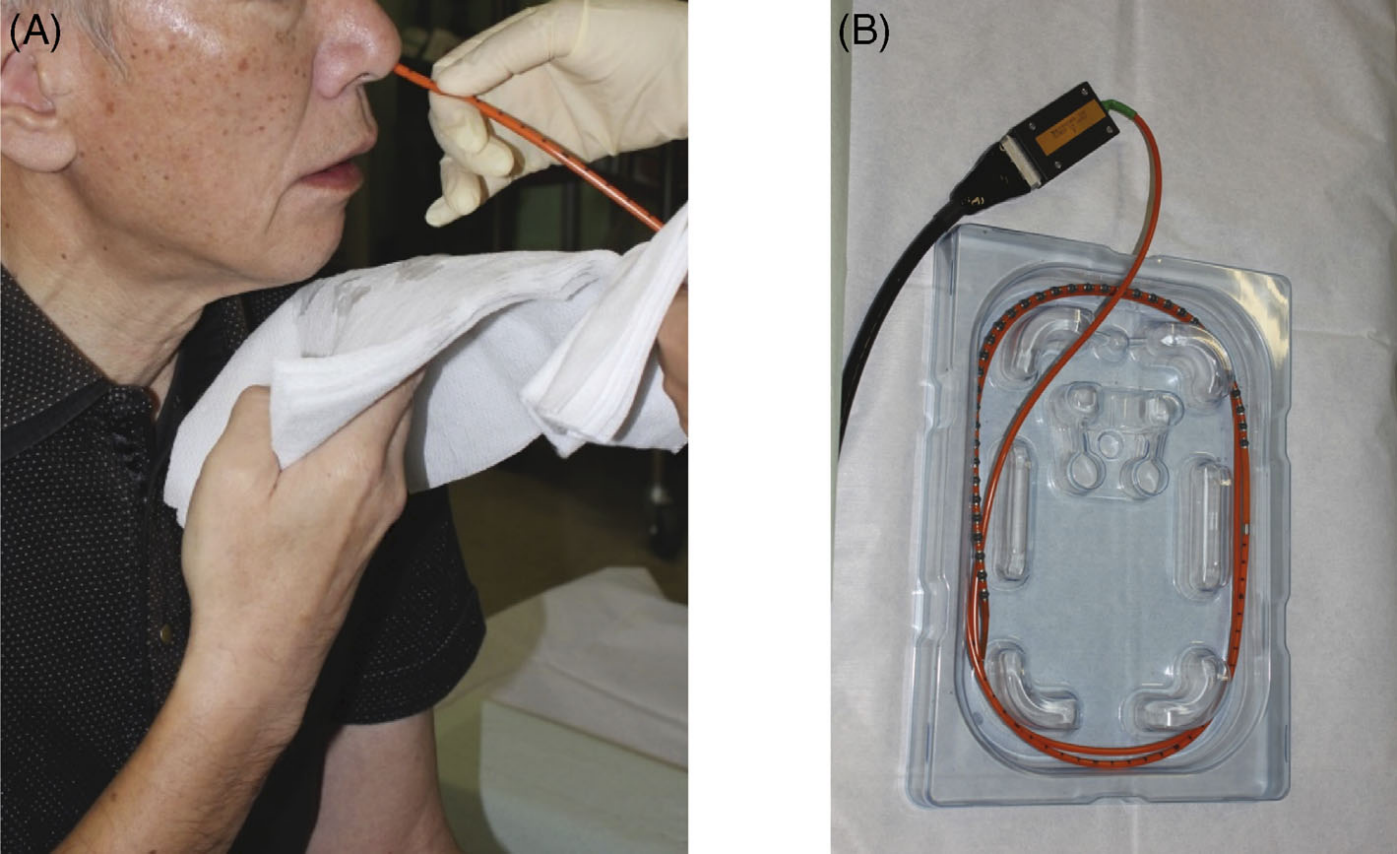
Esophageal high‐resolution manometry was conducted with Manoscan360 High‐Resolution Manometry System (Sierra Scientific Instruments, Los Angeles, CA). (A) Insertion of the catheter. (B) The catheter in the package; the 36 pressure sensors can be seen

**FIGURE 2:**
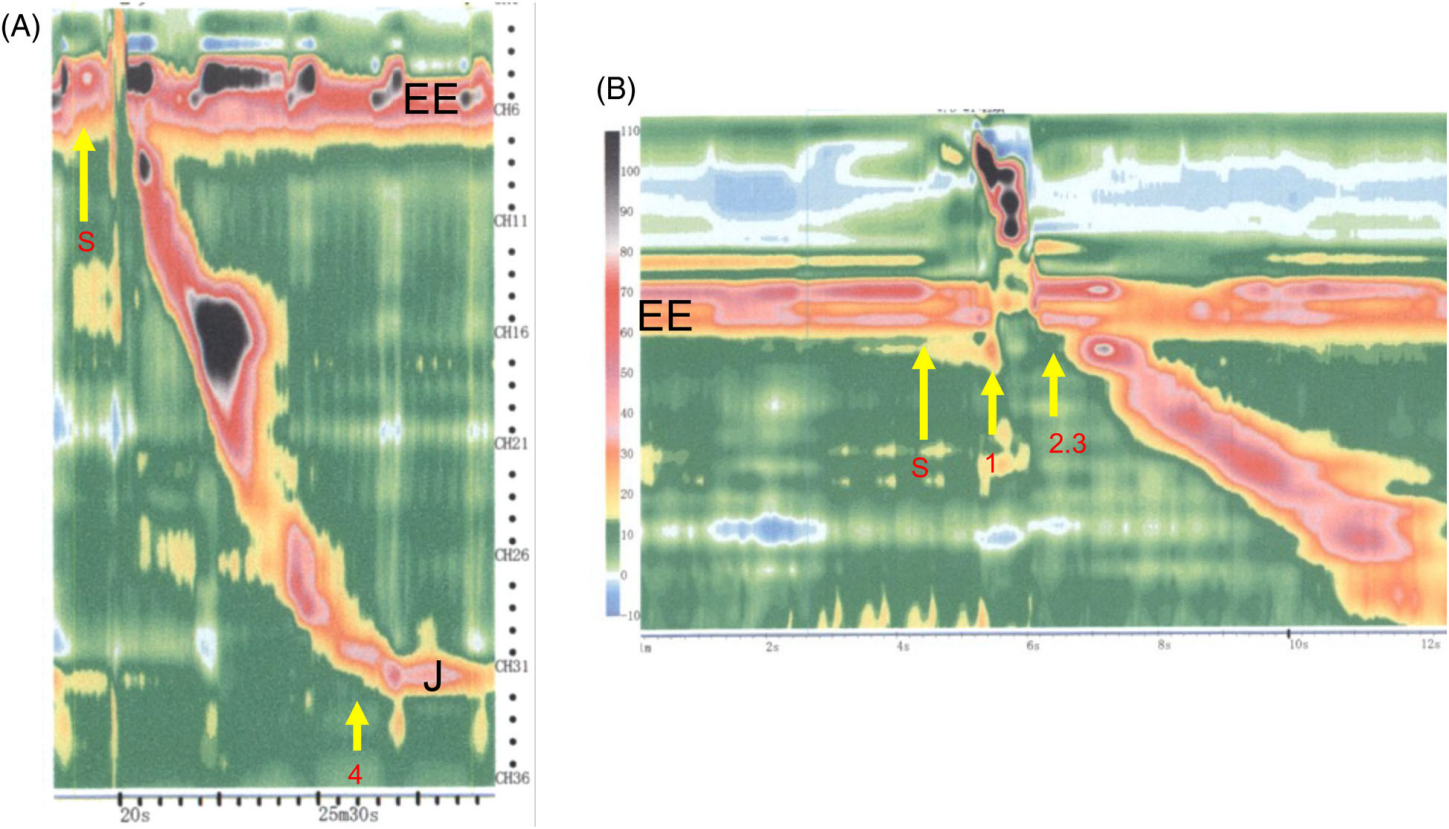
Pressure topography plots of the esophagus during a swallow by a healthy person. The four food passage‐related variables that were measured in the patients relative to the timepoint when swallowing movement started (set at 0 s as “S”) are indicated by the numbers 1–4. (A) and (B) show the following swallowing‐related timepoints and durations: #1) timepoint when hypopharyngeal pressure exceeds pressure at the esophageal entrance; #2) timepoint when the hypopharynx movement finishes; #3) timepoint when esophageal pressure exceeds esophagogastric junction pressure; and #4) timepoint when the esophageal peristalsis finishes. EE, esophageal entrance; J, esophagogastric junction

Esophageal manometry involves passing a manometry catheter through the nose into the stomach and asking the patient to consume small sips of water. The catheter bears 36 closely spaced pressure sensors that detect circumferential pressure changes in the esophagus lumen after a swallow. Manometry was conducted in all patients at least 3 months after surgery (unless the patient had a history of radiation therapy, in which case manometry was performed at least 6 months after surgery) with a high‐resolution manometer (Manoscan360 High‐Resolution Manometry System; Sierra Scientific Instruments, Los Angeles, CA) (Figure 1). The sensors on the catheter measure esophageal pressure changes during swallowing. When measuring esophageal peristalsis with this device, the effective pressure for food transportation was set to 20 mmHg/cm/s.^7^, ^8^ Figure 2A shows the pressure‐topography plot of the esophagus during a swallow by a healthy person and the four food passage‐related variables (numbered from 1 to 4 in chronological order during a single swallow) that were measured in our patients. All variables were measured relative to when swallowing movement started (set at 0 s). This timepoint was set for convenience because some tissues in the patients had been surgically resected. The four food passage‐related variables were timepoints when swallowing‐related events occurred, namely, when the hypopharyngeal pressure exceeded the pressure at the esophageal entrance (#1), when the hypopharynx movement finished (#2), when the esophageal pressure exceeded the esophagogastric junction pressure (#3), and when the esophageal peristalsis finished (#4) (Figure 2A,B).

### 2.5 MTF scoring

The MTF score is a simple tool for measuring swallowing ability.^9^, ^10^ It consists of three subscales that measure intake (M), time required for food intake (T), and the food consistency that the patient is able to ingest (F). All subscales are scored from 1 to 5, where 5 is normal and 1 is completely abnormal. Total MTF scores of 9–15, 7–8, and 3–6 signify good, acceptable, and poor swallowing ability, respectively (Table 1).

**TABLE 1:** MTF scoring system

MTF scoring system	
1. (M) Method of intake	Points
Tube feeding	1
Limited diet supported by tube feeding	2
Limited diet, pureed, or blenderized	3
Soft or semisolid diet	4
Normal diet	5
2. (T) Time of intake (minutes)	
About 50	1
About 40	2
About 30	3
About 20	4
About 10	5
3. (F) Food The types of food were classified into I–V: Calculate the total of the number of foods the patient can eat from groups I–V	
I. Liquid (e.g., water, tea, etc.)	
II. Liquid food (e.g., milk, smoothie, etc.)	
III. Semiliquid food (e.g., pudding, yogurt, etc.)	
IV. Soft food (e.g., risotto, rice gruel, etc.)	
V. Solid food	

*Note*: Calculate the sum of (M), (T), and (F). For example, it is necessary to process food in a blender and eat for 30 min. And in (F), if I, IV, and V cannot eat and only II and III are possible, the score will be 8 points. (M3T3F2).

### 2.6 Statistics

All data were expressed as mean and *SD* or *n* (%).

## 3 RESULTS

In total, 26 patients underwent esophageal or pharyngeal reconstruction during the 6.5‐year study period. Of these, 17 (65%) underwent postoperative esophageal manometry and were included in the study. The other patients did not undergo manometry testing because they did not wish to have additional tests.

### 3.1 Demographic, clinical, and treatment characteristics of the patients

The patients were on average 63.1 years old and 94% were male. The patients had advanced esophageal or pharyngeal cancer. A total of 13 underwent LPE and four underwent partial pharyngectomy + patch. Neoadjuvant or adjuvant radiotherapy was conducted in 12 patients. In seven patients, additional reconstruction was needed to manage postoperative complications (Table 2). The five healthy control subjects were on average 58.2 years old and all were male. None had any gastrointestinal illness (Table 3).

**TABLE 2:** Demographic and surgical characteristics of the17 patients

Variable	Mean or *n* (%)
Average age, years	63.1
Sex (male/female)	16/1 (94% male)
Larynx preserving surgery	17
LPE	13 (76.5%)
Partial pharyngectomy + patch	4 (23.5%)

Abbreviations: LPE, larynx‐preserving esophageal resection and microsurgical reconstruction.

**TABLE 3:** MTF scores and manometry results in the patients undergoing the five different types of surgery

	Normal control (*n =* 5)	LPE (*n =* 13)	PP + patch graft (*n =* 4)
Average age, years	58.2 (5.64)	63.8 (8.74)	61.3 (4.32)
Sex (male/female)	5/0	12/1	4/0
MTF score	13.8 (0.82)	9.84 (1.23)	11.25 (0.43)
Avg. time point (seconds)			
#1	1.41 (0.26)	2.45 (0.62)	1.66 (0.62)
#2	2.41 (0.27)	3.03 (0.75)	2.68 (0.54)
#3	2.41 (0.27)	—	2.68 (0.54)
#4	10.69 (1.29)	—	9.44 (0.29)
Evaluation period (postop. Months)	N/A	6.75 (1.23)	7.75 (1.47)

*Note*: All data are presented as mean (*SD*). Excludes cases for which valid data could not be collected. (1) Timepoint when hypopharyngeal pressure exceeds pressure at the esophageal entrance; (2) The finish point of hypopharynx movement; (3) Timepoint when esophageal pressure exceeds esophagogastric junction pressure; (4) The finish point of the esophageal peristalsis.

Abbreviations: LPE, larynx‐preserving esophageal resection and microsurgical reconstruction; PP, partial pharyngectomy.

### 3.2 Manometry data

Manometry was performed on average 7.3 (range, 6–10) months after surgery. At the time of testing, 19 patients reported never feeling dysphagia, five and four sometimes or frequently felt dysphagia, respectively, and one patient felt dysphagia all the time. This was supported by the MTF scores, which ranged from 7 to 12. The average MTF score was 9.9 and all patients had scores that fell into the acceptable (7–8) or good (9–15) swallowing range (Table 3).

The manometry data of 15 of the 17 patients were expressed as distance of the bolus from the oral entrance (cm) relative to time (seconds) after bolus consumption (Figure 2). The remaining two patients, who had undergone LPE, were unable to complete the test due to distress.

**FIGURE 3:**
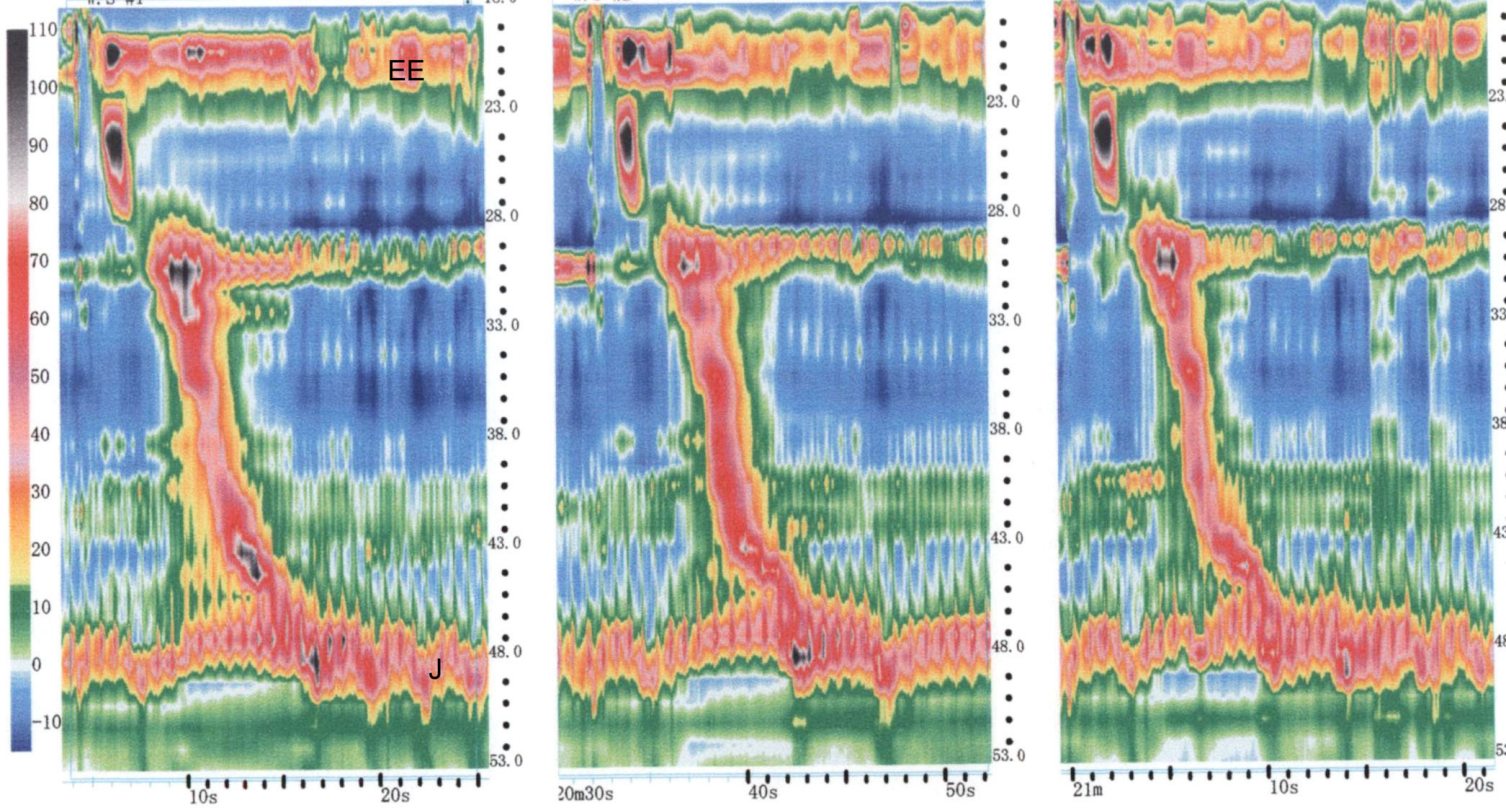
Typical esophageal manometry data of patients who underwent partial pharyngectomy and patch grafting. Peristaltic movement appears at the hypopharynx and progresses smoothly to the esophagus. EE, esophageal entrance; J, esophagogastric junction

**FIGURE 4:**
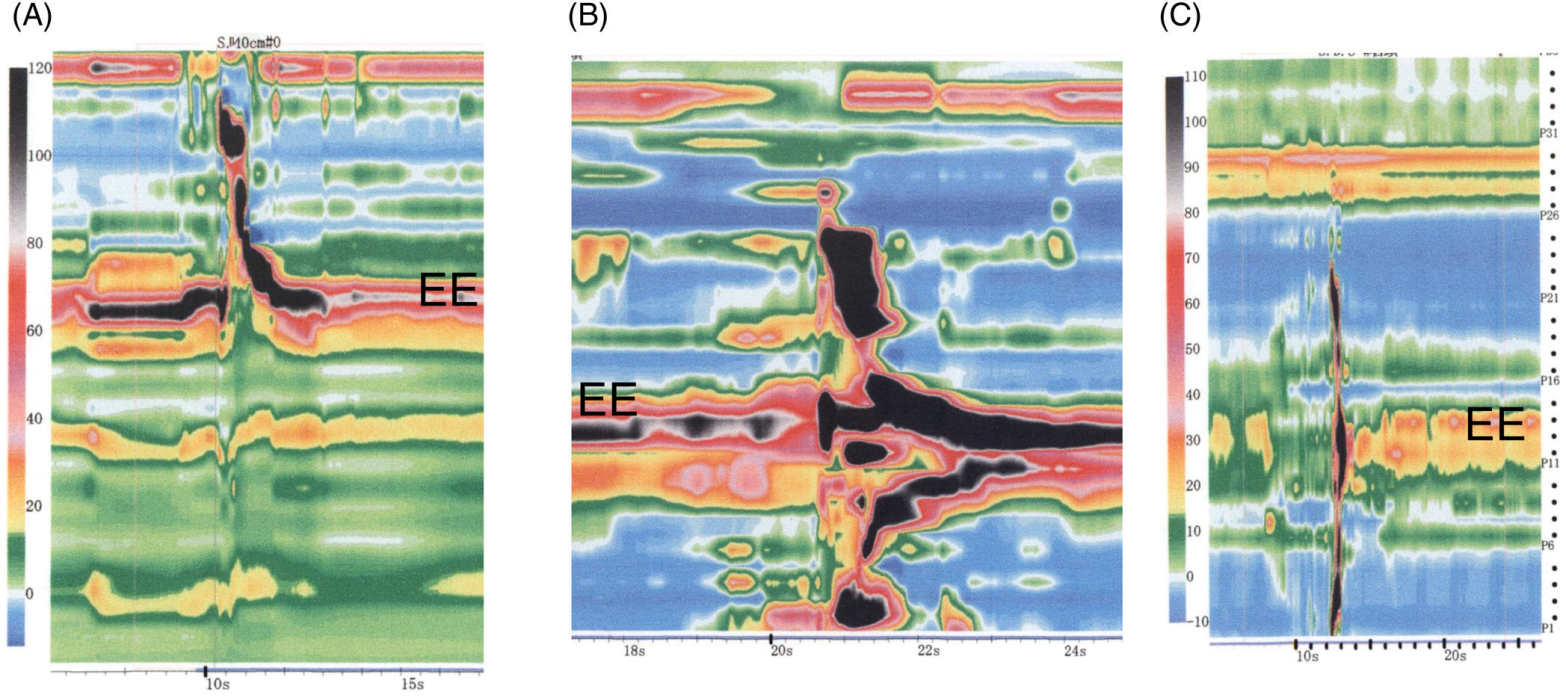
Esophageal manometry data of patients who underwent larynx‐preserving esophageal resection and microsurgical reconstruction (LPE). (A) shows typical manometry data of the LPE patients. Smooth swallowing pressure in the hypopharynx appears, but peristalsis has disappeared because the esophagus has been resected. (B, C) show the butterfly pattern manometry data of the two LPE patients with particularly low MTF scores. EE, esophageal entrance; J, esophagogastric junction

The manometry data of the controls and the patients who underwent the two types of surgery were examined separately. With regard to the five healthy controls, their average timepoints when the hypopharyngeal pressure exceeded pressure at the esophageal entrance (variable #1), the hypopharynx movement finished (variable #2), the esophageal pressure exceeded esophagogastric junction pressure (variable #3), and the esophageal peristalsis finished (variable #4) were 1.41, 2.41, 2.41, and 10.69 s, respectively (Table 3). The equivalent values for the four patients who had undergone partial pharyngectomy + patch were 1.66, 2.68, 2.68, and 9.44 s, respectively (Figure 3 and Table 3). LPE had marked effects on esophageal pressure dynamics after swallowing. Thus, in the 11 patients who had undergone LPE and completed manometry, the timepoint when hypopharyngeal pressure exceeded pressure at the esophageal entrance (variable #1) was slightly delayed (2.45 s) and it took longer to finish the hypopharynx movement (variable #2; 3.03 s). The timepoints when esophageal pressure exceeded esophagogastric junction pressure (variable #3) and esophageal peristalsis was completed (variable #4) could not be measured. (Figure 4A and Table 3).

The average MTF score of the LPE patients was slightly poorer than the partial pharyngectomy + patch patients (9.84 vs. 11.25). Notably, the two LPE patients who had particularly low MTF scores (8 points) had butterfly manometry patterns (Figure 4B,C).

## 4 DISCUSSION

### 4.1 Surgical approaches to head‐and‐neck and esophagus cancer resection and reconstruction

To assure oral intake after head‐and‐neck or esophageal cancer resection, thereby promoting good patient quality of life, it is essential to reconstruct the food passages to the stomach.^1^, ^2^, ^3^, ^4^, ^5^ Currently, the most favored reconstructive approach is to employ digestive organs such as the jejunum and stomach and/or skin flaps.^4^, ^11^, ^12^, ^13^ The reconstructive approach taken depends on which food‐passage organs have been resected. In the present study, the most common type of tumor‐resection surgery was LPE, where the larynx is preserved but the esophagus is resected. This was performed in cases of thoracic esophageal cancer that invaded into the cervical esophagus. Reconstruction was achieved with free‐jejunal transfer with gastric pull‐up or with pedicled intestine. In relation to the latter, the small intestine was often used and vascularization was required. Such pedicled vascularization is thought to prevent ischemia near the anastomosis in the cervix, thereby reducing the possibility of stenosis and fistula formation.^4^ All our LPE patients underwent endoscopic examination 7 days after surgery and then started dietary intake.

In four patients (14%), it was thought that their lesions could be controlled with partial resection of the hypopharynx. Since simple sutures were not possible, free‐tissue patch grafts had to be employed. In one case where the pharyngeal wall had to be reconstructed, the graft was from the jejunum. In the remaining three cases, a forearm flap was needed to reconstruct the complicated three‐dimensional structure of the arytenoid cartilages of the larynx. These pharyngectomy + patch patients underwent X‐ray videofluoroscopy examination 12 days after surgery and started dietary intake when there were no possibility of aspiration pneumonia or anastomosed intestinal leakage.

### 4.2 Effect of surgery on the dynamics of food transport

X‐ray videofluoroscopy is currently the most commonly used technique for visualizing food passage after surgery on the upper gastrointestinal tract because it effectively identifies passage obstructions and fistulas. However, it is less effective in determining the effect of surgery on the physiological dynamics of food transportation. To address this, we employed HRM, which is normally used in patients with reflux esophagitis and esophagus achalasia,^7^, ^8^, ^14^, ^15^, ^16^, ^17^ to determine the effects of the two types of surgery described above on physiological esophageal food‐passage functions. Specifically, we measured the effect of surgery on the timing of four chronological food‐passage events, starting with the increasing pharyngeal pressure and ending with completion of the esophageal movement. This is the first time esophageal high‐resolution manometry has been used to evaluate food‐passage outcomes after H&N‐E resection/reconstruction surgery.

As expected, the HRM pattern after partial hypopharyngectomy + patch was quite similar to that in the normal controls: the effective pressure increased in hypopharynx on average 1.66 s after swallowing movement started (vs. 1.41 s in the controls) and the effective esophageal peristalsis started at 2.68 s (vs. 2.41 s in the controls). This reflects the fact that this surgery does not reduce esophageal pressure. By contrast, LPE associated with poorer outcomes: it took 2.45 s before the effective pressure increased in the hypopharynx. Moreover, there was no internal pressure in the esophagus.

Notably, there were two LPE patients who showed an butterfly pattern in the pharynx on HRM (Figure 4B,C). Both had undergone reconstructive surgery for complications, and both had poor MTF scores (8 points). Hwang et al. have noted that this butterfly pattern occurs when the HRM catheter bends in a U‐shape: when they reinserted the catheter in a patient undergoing HRM after cardiological evaluation for chest pain, the butterfly pattern disappeared. Thus, our findings suggest that in our two patients, the catheter has become hooked in the esophagus due to the anatomical changes, thereby forming a U‐shaped bend with its end now lying near the cervical esophagus.^18^ This explains the poor MTF scores in these patients. It also supports the use of HRM after H&N‐E cancer surgery and reconstruction, since this technology will detect difficult food passage when, by contrast, fluoroscopy shows that there is no stenosis and fluid is passing through.

Thus, the HRM findings in our patients show that the type of surgery in esophageal reconstruction patients can have a marked impact on pharyngeal/esophageal food‐transport dynamics. In particular, if it eliminates the usual strong pressure due to esophageal peristalsis, the bolus must be propelled through the reconstruction into the stomach by gravity or slow and weak peristalsis. This delay could confuse patients and this in turn could induce the food to overflow into the hypopharynx, causing the pharynx to become clogged with food and provoking aspiration. It should be noted that although the hypopharyngectomy + patch group tended to be quite normal in terms of pharyngeal/esophageal food‐transport dynamics, there were also some differences from the healthy controls. Further studies will be needed to determine whether these differences in esophageal pressure and peristaltic movement reflect individual variation or true physiological changes.

## 5 CONCLUSIONS

In this study, patients underwent esophageal HRM after hypopharyngeal or esophageal cancer resection and reconstruction. This is the first time that esophageal HRM has been used in this setting. We showed that although fluoroscopy is excellent for visually assessing passage obstruction, esophageal HRM effectively captures surgery‐induced changes and deficiencies in esophageal pressure during swallowing. Although there are problems to be solved such as catheter reversal and patient distress, this study suggests that esophageal HRM could help to improve patient quality of life.

## CONFLICT OF INTEREST

The authors declare no conflicts of interest.

## AUTHOR CONTRIBUTION

Hiroki Umezawa: conception, design, analysis and interpretation of data, drafting and revising of the manuscript. Mariko Umezawa, Yu Hokazono, and Takeshi Matsutani: analysis and interpretation of data. Rei Ogawa: revising it for important intellectual content, final approval of the manuscript.

## Data Availability

The data that support the findings of this study are available from the corresponding author, Hiroki Umezawa, upon reasonable request.

## References

[1] Gurtner GC , Evans GR . Advances in head and neck reconstruction. Plast Reconstr Surg. 2000;106:672‐682. 10987478

[2] Ariyan S , Ross DA , Sasaki CT . Reconstruction of the head and neck. Surg Oncol Clin N Am. 1997;6:1‐15. 9031433

[3] Wong CH , Wei FC . Microsurgical free flap in head and neck reconstruction. Head Neck. 2010;32(9):1236‐1245. 20014446 10.1002/hed.21284

[4] Umezawa H , Nakao J , Matsutani T , Kuwahara H , Taga M , Ogawa R . Usefulness of the Clavien‐Dindo classification in understanding the limitations and indications of larynx‐preserving esophageal reconstruction. Plast Reconstr Surg Glob Open. 2016;4(11):e1113. 27975019 10.1097/GOX.0000000000001113 PMC5142485

[5] Lahtinen S , Koivunen P , Ala‐Kokko T , Kaarela O , Laurila P , Liisanantti JH . Swallowing‐related quality of life after free flap surgery due to cancer of the head and neck. Eur Arch Otorhinolaryngol. 2019;276(3):821‐826. 30593593 10.1007/s00405-018-05264-w PMC6411665

[6] Zenga J , Goldsmith T , Bunting G , Deschler DG . State of art: rehabilitation of speech and swallowing after total laryngectomy. Oral Oncol. 2018;86:38‐47. 30409318 10.1016/j.oraloncology.2018.08.023

[7] Yadlapati R . High‐resolution esophageal manometry: interpretation in clinical practice. Curr Opin Gastroenterol. 2017;33(4):301‐309. 28426462 10.1097/MOG.0000000000000369 PMC5568812

[8] Burgos‐Santamaría D , Marinero A , Chavarría‐Herbozo CM , Pérez‐Fernández T , López‐Salazar TR , Santander C . Normal values for water‐purfused esophageal high‐resolution manometry. Rev Esp Enferm Dig. 2015;107(6):354‐358. 26031863

[9] Fujimoto Y , Hasegawa Y , Yamada H , Ando A , Nakashima T . Swallowing function following extensive resection of oral or oropharyngeal cancer with laryngeal suspension and cricopharyngeal myotomy. Laryngoscope. 2007;117(8):1343‐1348. 17585279 10.1097/MLG.0b013e3180686590

[10] Fujimoto Y , Matsuura H , Kawabata K , Takahashi K , Tayama N . Assessment of swallowing ability scale for oral and oropharyngeal cancer patients. Nihon Jibiinkoka Gakkai Kaiho. 1997;100(11):1401‐1407. 9423324 10.3950/jibiinkoka.100.1401

[11] Doki Y , Okada K , Miyata H , et al. Long‐term and short‐term evaluation of esophageal reconstruction using the colon or the jejunum in esophageal cancer patients after gasterectomy. Dis Esophagus. 2008;21:132‐138. 18269648 10.1111/j.1442-2050.2007.00738.x

[12] Kadota H , Sakuraba M , Kimata Y , Hayashi R , Ebihara S , Kato H . Larynx‐preserving esoph‐ agectomy and jejunal transfer for cervical esophageal carcinoma. Laryngoscope. 2009;119:1274‐1280. 19444878 10.1002/lary.20493

[13] Lewin JS , Barringer DA , May AH . Functional outcomes after laryngopharyngectomy with anterolateral thigh flap reconstruction. Head Neck. 2006;28:142‐149. 16302192 10.1002/hed.20308

[14] Ivashkin VT , Maev IV , Trukhmanov AS , et al. High resolution manometry and new classification of esophageal motility disorders. Ter Arkh. 2018;90(5):93‐100. 30701897 10.26442/terarkh201890593-100

[15] Aggarwal N , Lopez R , Gabbard S , Wadhwa N , Devaki P , Thota PN . Spectrum of esophageal dysmotility in systemic sclerosis on high‐resolution esophageal manometry as defined by Chicago classification. DisEsophagus. 2017;30(12):1‐6. 10.1093/dote/dox067 28881879

[16] Patel A , Posner S , Gyawali CP . Esophageal high‐resolution manometry in gastroesophageal reflux disease. JAMA. 2018;320(12):1279‐1280. 30128486 10.1001/jama.2018.8694

[17] Bredenoord AJ , Fox M , Kahrilas PJ , Pandolfino JE , Schwizer W , Smout AJ . International high resolution manometry working group. Chicago classification criteria of esophageal motility disorders defined in high resolution esophageal pressure tomography. Neurogastroenterol Motil. 2012;24(1):57‐65. 22248109 10.1111/j.1365-2982.2011.01834.x PMC3544361

[18] Hwang JK , Hong SG , Joo MK , Park JJ , Kim JS , Bak YT . Butterfly in the esophagus: what is wrong? J Neurogastroenterol Motil. 2010;16(1):94‐95. 20535333 10.5056/jnm.2010.16.1.94 PMC2879826

